# Bridging the Digital Prevention Gap: A Systematic Literature Review of Digital Health Literacy and Preventive Health Behaviors in Low-Income Communities

**DOI:** 10.7759/cureus.112767

**Published:** 2026-07-16

**Authors:** Tejinder Singh Ajmani, Somya Pareek, Karthika Padmavathy, Pranjal Upadhayay, Divyangkumar Patel, Bikramaditya Mukherjee

**Affiliations:** 1 Department of Anaesthesia, Gandhi Medical College, Madhya Pradesh Medical Science University (MPMSU), Jabalpur, IND; 2 Department of Pathology, Sri Lalithambigai Medical College and Hospital, Dr. MGR Educational and Research Institute, Chennai, IND; 3 Department of Public Health and Medical Education, National Medical College and Teaching Hospital, Birgunj, NPL; 4 Department of Community Medicine, Dr. ND Desai Faculty of Medical Science and Research, Dharmsinh Desai University, Nadiad, IND; 5 Department of Biochemistry, KPC Medical College and Hospital, Jadavpur, IND

**Keywords:** digital divide, digital health literacy, health equity, low-income communities, preventive health behavior

## Abstract

Digital health literacy is increasingly central to preventive healthcare, particularly in low-income and socioeconomically vulnerable communities where limited internet access, lower health literacy, and digital exclusion may restrict engagement with prevention services. Although digital health tools are expanding, evidence remains fragmented on how digital health literacy is associated with preventive behaviors in disadvantaged populations. This systematic literature review examined the association between digital health literacy and preventive health behaviors, including COVID-19 prevention, vaccination intention, online health information seeking, screening-related decision support, and chronic disease self-management. A structured search of electronic databases was conducted using terms related to digital health literacy, eHealth literacy, preventive health behavior, socioeconomic vulnerability, and the digital divide. Eligible primary studies assessed digital or eHealth literacy or online health information-related skills and reported prevention-related outcomes. One measurement-support study was retained only to contextualize the digital health literacy assessment and was not included in the preventive outcome synthesis. Findings were synthesized narratively because of heterogeneity in study design, populations, measures, and outcomes. Higher digital health literacy was generally associated with better prevention-related outcomes, especially COVID-19 preventive behaviors, vaccination intention, and online health information use. Education, income, internet access, computer literacy, employment, and general health literacy repeatedly influenced digital health literacy. The evidence also suggested that vulnerable users can benefit from digital prevention tools when interventions include accessible design, low-literacy content, privacy safeguards, and user support. The digital prevention gap is multidimensional, reflecting both individual skill differences and structural barriers. Strengthening digital health literacy should be treated as a core strategy for equitable prevention, not merely as a technology-access issue.

## Introduction and background

Preventive health behavior is essential for reducing avoidable morbidity, particularly in low-income and socioeconomically vulnerable settings where limited healthcare access, low educational attainment, poverty, and unreliable digital connectivity may restrict timely prevention. Health systems increasingly deliver prevention messages through websites, mobile applications, patient portals, telemedicine platforms, and social media channels, making access to digital health information an important public health issue [1]. Digital health literacy, originally conceptualized within the eHealth literacy framework, refers to the ability to seek, find, understand, and appraise health information from electronic sources and apply the knowledge gained to address or solve a health problem [2]. In this review, digital health literacy is treated as the central exposure, whereas internet access, digital equity, health literacy, and socioeconomic status are considered contextual determinants that may influence how digital health information is accessed and used.

Digital health literacy extends beyond basic device use. It includes information searching, comprehension, appraisal of credibility, relevance assessment, privacy awareness, trust, and application of online health information to decision-making [3-5]. These skills are directly relevant to preventive health behavior because prevention often requires individuals to identify reliable information, distinguish it from misinformation, understand personal risk, and apply guidance related to vaccination, screening, infection prevention, or chronic disease self-management [6]. Global evidence indicates that digital health inequalities, including barriers related to connectivity, affordability, usability, language, privacy, digital confidence, and trust, may restrict the use of online health information for preventive health decisions in socioeconomically vulnerable populations [3].

The digital prevention gap can be understood as the difference between making digital prevention tools available and ensuring that vulnerable users can meaningfully use them. Individual factors such as age, education, income, general health literacy, language, disability, and previous technology experience may affect digital health literacy [7,6]. Community and health system factors, including broadband availability, device affordability, platform design, integration with healthcare services, and institutional trust, may further shape whether digital health information can be used for prevention [8,6]. These factors are included in this review only as determinants or barriers related to digital health literacy, rather than as separate outcomes of digital divide or digital equity research.

The relevance of digital health literacy is evident across prevention settings. In chronic disease prevention and self-management, patients may need to use online information to understand medications, lifestyle changes, monitoring, complication prevention, and follow-up care [9,10]. In primary care, digital tools may support screening reminders, risk assessment, patient education, lifestyle counselling, and self-management, but their practical value depends on whether underserved populations can access, understand, evaluate, and use the information provided [11]. In low- and middle-income countries, low literacy, rural residence, limited connectivity, and cost of access may restrict the use of telemedicine and mobile health applications for prevention [9].

Recent literature has addressed digital health literacy, digital health equity, telemedicine access, and digital health interventions separately. Several reviews have focused on broad digital health inequalities, health equity, or digital intervention implementation, rather than specifically synthesizing how digital health literacy relates to preventive health behaviors in low-income or socioeconomically vulnerable communities [3,6,8,12]. The present review differs by focusing on digital health literacy as the core construct and preventive health behavior as the outcome of interest, with socioeconomic and digital access factors examined as contextual determinants. This focus is important because access to a digital tool alone does not ensure the ability to interpret information, assess credibility, protect privacy, trust guidance, or act on prevention recommendations.

Objectives of the review

This systematic literature review, therefore, aimed to examine the association between digital health literacy and preventive health behaviors in low-income and socioeconomically vulnerable communities. It also assessed how socioeconomic factors, digital access, and information appraisal skills relate to digital health literacy and explored barriers and facilitators affecting the use of digital health information for prevention in disadvantaged populations.

## Review

Materials and methods

Study Design

This systematic literature review aimed to assess the relationship between digital health literacy and preventive health behaviors in low-income, socioeconomically disadvantaged, or vulnerable populations. The review was conducted according to the Preferred Reporting Items for Systematic Reviews and Meta-Analyses (PRISMA) 2020 guidelines. The review protocol was not prospectively registered in the International Prospective Register of Systematic Reviews (PROSPERO) or any other systematic review registry. The literature search covered articles published from January 2015 to June 2026, and the final search was completed on June 10, 2026. Because of methodological differences across the included studies, a narrative synthesis was conducted, and no meta-analysis was performed.

Research Question

The review was guided by the following research question: How is digital health literacy associated with preventive health behaviors among low-income or socioeconomically disadvantaged communities?

Search Strategy

A structured literature search was conducted in four electronic databases: PubMed/MEDLINE, Scopus, Google Scholar, and Web of Science. The search included studies addressing digital health literacy, eHealth literacy, online health information seeking, preventive health behaviors, vaccination intention, and socioeconomic disadvantage. Search terms were combined using Boolean operators and included "digital health literacy", "eHealth literacy", "electronic health literacy", "online health information", "health information seeking", "preventive health behaviour", "COVID-19 prevention", "vaccination intention", "vaccine uptake intention", "low-income", "socioeconomic status", "vulnerable population", "underserved population", "digital divide", and "health equity". The complete database-specific search strategy is summarized in Table 1.

**Table 1:** Search strategy

Database	Search terms and Boolean strategy
PubMed/MEDLINE	**(("Health Literacy"[Mesh] OR "digital health literacy"[tiab] OR "eHealth literacy"[tiab] OR "electronic health literacy"[tiab] OR eHEALS[tiab] OR DHLI[tiab] OR "digital literacy"[tiab] OR "online health information"[tiab] OR "health information seeking"[tiab] OR "internet health information"[tiab]) AND ("Preventive Health Services"[Mesh] OR "Health Behavior"[Mesh] OR "Health Promotion"[Mesh] OR "preventive health behavio*"[tiab] OR "preventive behavio*"[tiab] OR prevention[tiab] OR screening[tiab] OR vaccination[tiab] OR immunization[tiab] OR immunisation[tiab] OR "vaccine uptake"[tiab] OR "vaccination intention"[tiab] OR "physical activity"[tiab] OR diet[tiab] OR nutrition[tiab] OR smoking[tiab] OR "substance use"[tiab] OR "chronic disease self-management"[tiab]) AND ("Socioeconomic Factors"[Mesh] OR "Poverty"[Mesh] OR "Vulnerable Populations"[Mesh] OR "Medically Underserved Area"[Mesh] OR "low income"[tiab] OR low-income[tiab] OR "socioeconomic status"[tiab] OR "socioeconomic disadvantage"[tiab] OR underserved[tiab] OR vulnerable[tiab] OR disadvantaged[tiab] OR "low-resource"[tiab] OR "digital divide"[tiab] OR "health equity"[tiab]))
Scopus	("digital health literacy" OR "eHealth literacy" OR "electronic health literacy" OR eHEALS OR DHLI OR "online health information" OR "health information seeking") AND ("preventive health behavior" OR "preventive health behaviour" OR "COVID-19 prevention" OR "vaccination intention" OR "vaccine uptake intention" OR screening OR "chronic disease self-management") AND ("low-income" OR "socioeconomic status" OR "socioeconomic disadvantage" OR underserved OR vulnerable OR "digital divide" OR "health equity")
Google Scholar	("digital health literacy" OR "eHealth literacy" OR "electronic health literacy" OR eHEALS OR DHLI OR "digital literacy" OR "online health information" OR "health information seeking") AND ("preventive health behavior" OR "preventive health behaviour" OR prevention OR screening OR vaccination OR "vaccination intention" OR "vaccine uptake" OR "physical activity" OR diet OR nutrition OR smoking OR "substance use" OR "chronic disease self-management") AND ("low income" OR low-income OR underserved OR vulnerable OR disadvantaged OR "socioeconomic status" OR "socioeconomic disadvantage" OR "low-resource" OR "digital divide" OR "health equity")
Web of Science	("digital health literacy" OR "eHealth literacy" OR "electronic health literacy" OR eHEALS OR DHLI OR "online health information" OR "health information seeking") AND ("preventive health behavior" OR "preventive health behaviour" OR "COVID-19 prevention" OR "vaccination intention" OR "vaccine uptake intention" OR screening OR "chronic disease self-management") AND ("low-income" OR "socioeconomic status" OR "socioeconomic disadvantage" OR underserved OR vulnerable OR "digital divide" OR "health equity")

Eligibility Criteria

Eligible studies included cross-sectional studies, cohort studies, qualitative studies, mixed-methods studies, and intervention or usability studies if they evaluated digital health literacy, eHealth literacy, or online health information-related skills and reported a prevention-related outcome, such as health information seeking, vaccination intention, screening-related decision-making, preventive behavior, or chronic disease self-management. Studies were required to focus on low-income, socioeconomically disadvantaged, vulnerable, underserved, or digitally marginalized populations. Reporting socioeconomic variables alone, such as income or education, was not considered sufficient unless the population was clearly described as disadvantaged or vulnerable. Measurement-validation studies were excluded from the preventive outcome synthesis. One measurement-support study was retained only to contextualize eHealth literacy measurement and was not used as evidence for preventive behavior outcomes. Only full-text English-language articles were included. The English-language restriction was acknowledged as a limitation of the review.

Studies were excluded if they were systematic reviews, scoping reviews, narrative reviews, editorials, commentaries, conference abstracts, protocols, or background papers. Studies were also excluded if they did not assess digital health literacy, eHealth literacy, or online health information-related skills; did not report a prevention-related outcome; did not focus on low-income, socioeconomically disadvantaged, vulnerable, underserved, or digitally marginalized populations; were not available as full-text English-language articles; or lacked extractable data relevant to the review objectives. Measurement-validation studies were excluded from the preventive outcome synthesis unless retained only as measurement-support evidence for interpreting digital/eHealth literacy assessment.

Study Selection

All identified records were screened in stages. Duplicate records were removed first. Titles and abstracts of peer-reviewed journal articles published from January 2015 to June 2026 were screened against the eligibility criteria. The final search was completed on June 10, 2026. Full-text peer-reviewed primary studies were then assessed for eligibility. Conference abstracts, preprints, grey literature, protocols, editorials, commentaries, review articles, methodological papers, and background papers were excluded from the outcome synthesis. Only eligible primary studies reporting digital/eHealth literacy and prevention-related outcomes were included in the final synthesis. Background, conceptual, and methodological references were used only to support the Introduction, Methods, or Discussion and were not counted as included primary studies.

Data Extraction

Study screening and data extraction were performed independently by two reviewers using predefined eligibility criteria and a structured extraction form. Extracted variables included author name, publication year, country or setting, study design, sample size, population characteristics, digital health literacy measure, preventive health outcome, key numerical results, and major conclusions. Findings related to socioeconomic status, digital access, health literacy, internet use, and barriers to digital prevention were also extracted. Any disagreement during study screening or data extraction was resolved through discussion, and unresolved differences were settled by consultation with a third reviewer. 

Risk-of-Bias Assessment

Risk of bias was assessed independently by two reviewers using design-specific tools. For cross-sectional studies, the Newcastle-Ottawa Scale (NOS) was used as the original methodological basis [13], and the cross-sectional adaptation was guided by the approach described by Herzog et al. [14]. The assessed domains included participant selection, comparability or adjustment for confounding, and ascertainment of exposure and outcome. Studies were categorized as low, moderate, or high risk of bias based on predefined domain-level criteria and the severity of methodological concerns. Disagreements between the two reviewers were resolved through discussion, and unresolved differences were settled by consultation with a third reviewer. For non-cross-sectional studies, the risk of bias was assessed according to the study design. The qualitative study was assessed using the Critical Appraisal Skills Programme (CASP) Qualitative Checklist [15]. The study conducted within a randomized trial framework was assessed using the revised Cochrane Risk of Bias tool for randomized trials (RoB 2), rather than being assessed only as a usability-focused study [16]. Measurement-validation studies were not included in the main outcome synthesis and were therefore excluded from the main risk-of-bias assessment. Overall appraisal was graded as low risk when all or most domains showed no major concerns, moderate risk when one or more domains showed some concerns without a critical limitation, and high risk when major concerns were present in key domains such as participant selection, confounding control, exposure or outcome measurement, or completeness of reporting.

Data Synthesis

Due to the diversity of populations, study designs, digital health literacy tools and measures, and prevention-related outcomes in the included studies, a narrative synthesis was performed. The results have been clustered across three broad themes: digital health literacy and preventive health behaviors, socioeconomic/digital access factors, and barriers and facilitators to digital prevention among vulnerable populations. Where available, numerical results (adjusted ORs, regression coefficients, proportions) were summarized.

Results

Search Results and Study Selection

**Figure 1:**
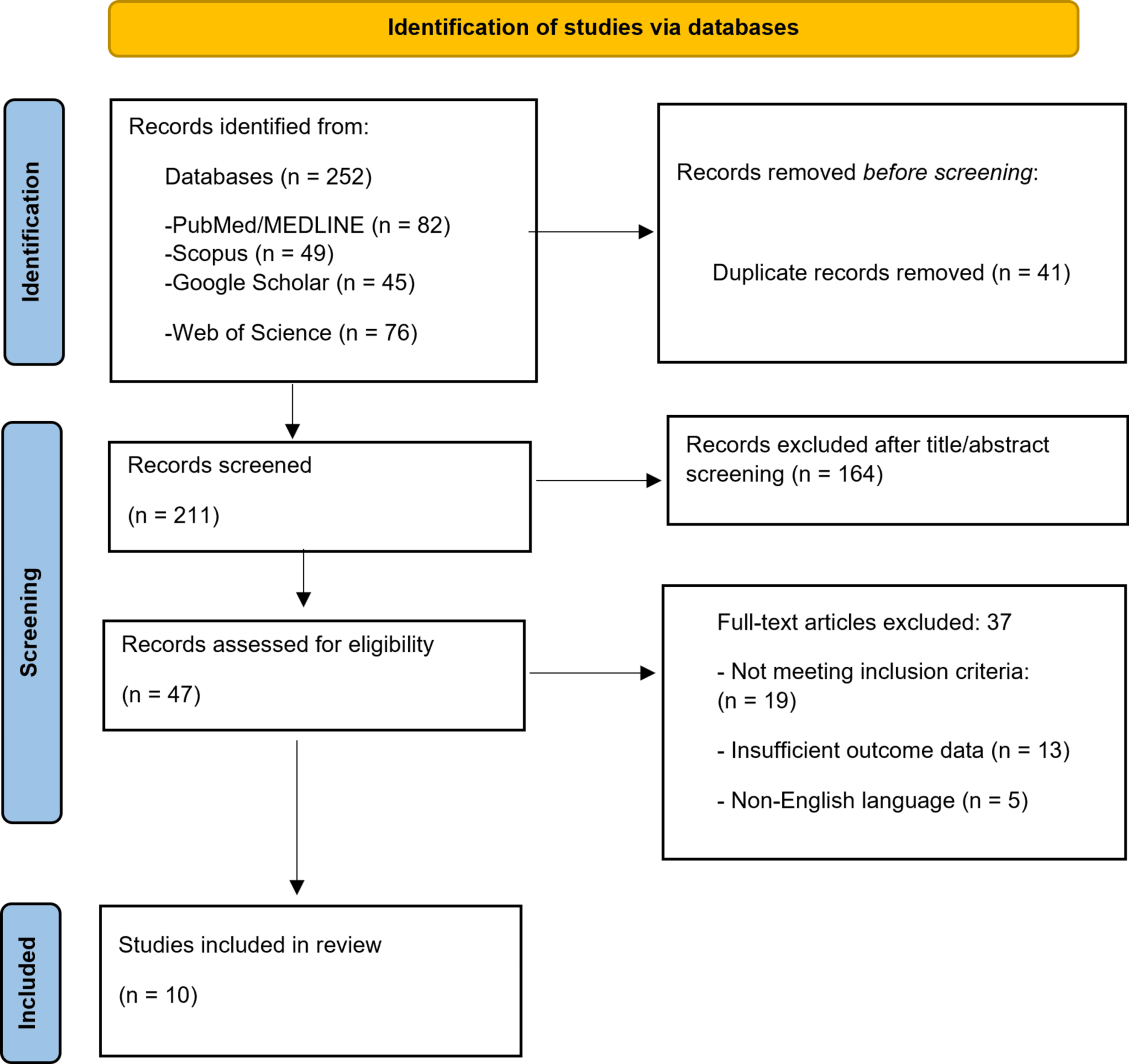
PRISMA flow diagram of study selection PRISMA: Preferred Reporting Items for Systematic Reviews and Meta-Analyses

The database search identified 252 records, including 82 from PubMed/MEDLINE, 49 from Scopus, 45 from Google Scholar, and 76 from Web of Science. After removing 41 duplicate records, 211 records were screened by title and abstract. Of these, 164 records were excluded because they were not relevant to the review question or did not meet the preliminary eligibility criteria. A total of 47 full-text articles were assessed for eligibility. During full-text review, 37 articles were excluded: 19 did not meet the inclusion criteria, 13 had insufficient outcome data, and five were excluded because they were not published in English. Finally, 10 reports were included in the review, including nine reports contributing directly to the outcome synthesis and one measurement-support report retained only to contextualize eHealth literacy measurement. The study selection process is shown in Figure 1, which specifies the databases searched and the number of records identified from each database.

Study Characteristics

The included reports were published from 2015 to 2023 and were selected because they addressed populations or subgroup findings relevant to socioeconomic vulnerability, underserved healthcare access, or digital marginalization. These populations included lower-income B40 adults in Malaysia, chronic disease patients in Ethiopia, safety-net patients and caregivers in the United States, vulnerable primary-care patients due for colorectal cancer screening, young adults in Bangladesh, and community samples in which deprivation, low income, limited health literacy, or restricted internet access were specifically assessed. Studies from broader populations, including Hong Kong, South Korea, England, and the multi-country survey, were retained only when they reported vulnerability-related determinants or subgroup findings, such as socioeconomic status, education, income, residence, health literacy, or digital access. Cross-sectional survey designs were most common. Sample sizes ranged from 16 participants in a qualitative safety-net portal study to 4700 participants in a multi-country study on digital health literacy and vaccination intention.

The primary digital health literacy tools used were the eHealth Literacy Scale (eHEALS), adapted eHealth literacy measures, and the Digital Health Literacy Instrument (DHLI). Preventive outcomes most commonly involved COVID-19 preventive behaviors and vaccination intention. Other prevention-related outcomes included online health information seeking, chronic disease self-management support, and colorectal cancer screening decision support. The characteristics of the included studies are summarized in Table 2 using the Population, Exposure, Comparator/Comparison, Outcome (PEO)/Population, Exposure, Outcome (PECO) framework, which is more appropriate for observational studies examining exposure-outcome associations.

**Table 2:** Study characteristics using the PEO/PECO framework B40: bottom 40% income group; COVID-19: coronavirus disease 2019; DHLI: Digital Health Literacy Instrument; eHEALS: eHealth Literacy Scale; eHealth: electronic health; N: sample size; PECO: Population, Exposure, Comparator/Comparison, Outcome; PEO: Population, Exposure, Outcome; UAE: United Arab Emirates

Study	Setting/population	Design/sample	Population (P)	Exposure (E)	Comparator/comparison (C), if applicable	Outcome (O)	Key findings
Guo et al. [17]	Hong Kong adults during COVID-19	Cross-sectional survey; N=1501	Adults aged ≥18 years in Hong Kong during the COVID-19 pandemic	eHealth literacy measured using the Chinese eHEALS and web-based COVID-19 information seeking	Participants with lower versus higher eHEALS scores; participants who sought versus did not seek web-based COVID-19 information; socioeconomic subgroups by education, income, age, employment, and chronic disease status	Mask wearing, handwashing, social distancing, and household drainage hygiene	Higher education and income were associated with higher eHealth literacy and more online COVID-19 information seeking. Higher eHEALS scores were associated with greater adherence to several COVID-19 preventive behaviors
Marzo et al. [18]	Lower-income B40 adults in Selangor, Malaysia	Cross-sectional telephone survey; N=381	Lower-income B40 adults aged ≥18 years living in Selangor, Malaysia	Adapted DHLI domains, including information searching, content generation, evaluating reliability, determining relevance, and protecting privacy	Digital health literacy differences across demographic and socioeconomic groups, including age, sex, marital status, education, employment status, and household income	COVID-19-related information seeking and interpretation relevant to prevention	Most respondents reported that COVID-19 information searching was easy, but difficulties remained in evaluating reliability, creating content, and protecting privacy. Less educated, unemployed, and widowed groups were identified as needing additional support
Shiferaw et al. [19]	Chronic disease patients in Ethiopia	Institutional cross-sectional survey; 423 approached; 95.3% response rate	Internet user chronic disease patients attending follow-up care at the University of Gondar Comprehensive Specialized Hospital, Ethiopia	eHealth literacy measured using eHEALS	Patients with high versus low eHealth literacy; subgroups by education, occupation, residence, income, internet use frequency, computer literacy, knowledge of online resources, and attitude toward online resources	Health information use for chronic disease management and prevention-related decision-making	eHealth literacy was relatively low; 46.5% had high eHealth literacy. Higher education, income, computer literacy, daily internet use, positive attitudes toward online resources, and knowledge of online health resources predicted higher eHealth literacy
Tieu et al. [20]	Safety-net patients and caregivers in the United States	Qualitative study; 16 interviews	Patients with chronic disease and caregivers in a US safety-net healthcare system, including participants with limited health literacy	Portal readiness, health literacy, technical skill, and privacy concerns affecting willingness to use an online patient portal	Participants with limited versus adequate health literacy; patient versus caregiver perspectives; participants with different levels of technical skill, internet access, and privacy concerns	Chronic disease self-management and portal-supported care behaviors	Participants were generally interested in portal use, but barriers included limited technical skill, limited health literacy, security concerns, reading/typing difficulty, and preference for in-person communication. Caregivers viewed portals as useful for interpreting information and managing care
Miller et al. [21]	Vulnerable primary-care patients in the United States due for colorectal cancer screening	Usability analysis within randomized trial; N=450	Primary-care patients aged 50-74 years who were due for colorectal cancer screening, including vulnerable participants with limited health literacy and low income	Use of the mPATH mobile health iPad program, including a self-survey and either a colorectal cancer screening decision aid or a health behavior video	Vulnerable versus non-vulnerable participants; participants who required assistance versus those who completed the program independently	Colorectal cancer screening decision support	Most vulnerable participants completed a low-literacy iPad decision-aid program. Design features included large buttons, audio narration, simple navigation, and low reading-level content
Paige et al. [22]	US adults across age groups	Measurement-invariance study; N=829	US adults across the lifespan, including Millennials, Generation X, Baby Boomers, and the Silent Generation	Electronic health literacy measured using eHEALS	Age group comparisons across generational cohorts and comparison of eHEALS factor structure	Measurement-support evidence; no direct preventive behavior outcome	eHEALS showed multidimensional measurement properties across age groups, including awareness, information seeking, and engagement/evaluation. This study was retained only to support the interpretation of digital/eHealth literacy measurement and was not included in the preventive outcome synthesis
Estacio et al. [23]	Stoke-on-Trent, England; community sample	Cross-sectional survey; N=1046	Community adults in Stoke-on-Trent, England, participating in a city-wide health literacy survey	Health literacy, internet access, and internet use for seeking health information	Respondents with low, marginal, or adequate health literacy; subgroups by age, education, income, perceived health, social isolation, ethnicity, and deprivation	Online health information seeking	Adequate health literacy was strongly associated with internet access. Older age, lower education, lower income, poor perceived health, and social isolation predicted lower internet access and health information use
Choi and Lee [24]	South Korean young adults aged 20-39 years	Cross-sectional study; N=324	South Korean young adults aged 20-39 years who used the internet through computers, tablets, or mobile phones	COVID-19-related eHealth literacy measured using adapted eHEALS, together with Health Belief Model constructs and self-efficacy	Participants with different levels of COVID-19-related eHealth literacy, perceived susceptibility, perceived severity, perceived benefits, perceived barriers, and self-efficacy	COVID-19 preventive behaviors	COVID-19-related eHealth literacy and self-efficacy were significant positive predictors of preventive behaviors. The model explained 23.4% of the variance in preventive behavior
Marzo et al. [25]	Adults from 53 countries, with major representation from Malaysia, the Philippines, Turkey, the UAE, Indonesia, and other countries	Internet-based cross-sectional survey; N=4700	Adults aged ≥18 years from 53 countries who completed an internet-based survey on COVID-19 vaccination	DHLI domains: information searching, content generation, evaluating reliability, and determining relevance	Participants with sufficient versus limited digital health literacy; subgroups by country, residence, education, employment, and income	Intention to support COVID-19 vaccination	Sufficient digital health literacy was associated with higher vaccination intention. Urban residence, education, employment, and income were associated with vaccination intention. The ability to assess the relevance of online information was particularly important
Nath et al. [26]	Young adults in Bangladesh, a lower-middle-income country	Cross-sectional study; N=343	Young adults aged 18-30 years in Bangladesh	eHealth literacy measured using eHEALS, vaccine literacy, and vaccine hesitancy domains	Participants with different levels of eHealth literacy, vaccine literacy, and vaccine hesitancy; models adjusted for age, sex, opinion-leader influence, COVID-19 experience, and conspiracy-belief variables	COVID-19 vaccine uptake intention	Vaccine hesitancy was the strongest predictor of vaccine uptake intention. eHealth literacy was positively associated with vaccine uptake intention, while vaccine literacy was not significantly associated

Synthesis of Key Findings

Across the included studies, digital health literacy appeared to relate to preventive health behaviors through three recurring areas: access to online health information, appraisal of information reliability and relevance, and confidence in using digital information for health decisions [17-21,23-26]. These areas were most clearly reported in studies on COVID-19 preventive behaviors, vaccination intention, online health information seeking, screening decision support, and chronic disease self-management [17-21,23-26]. Vulnerability-related factors, including lower income, lower education, unemployment, rural residence, limited health literacy, restricted internet access, and lower technical ability, were also reported across several included studies [17-21,23,25,26]. Evidence was strongest for COVID-19 prevention and vaccination intention, whereas fewer studies addressed cancer screening, chronic disease prevention, and non-pandemic health promotion. Since most included studies were cross-sectional and some assessed intention rather than observed behavior, these findings should be interpreted as associations rather than causal pathways.

Risk-of-Bias Assessment

The risk-of-bias assessment showed an overall moderate risk of bias across the included studies. Ratings were assigned using the predefined grading approach described in the Methods section. Most cross-sectional studies described their populations clearly and used appropriate digital/eHealth literacy measures, but common concerns included cross-sectional design, convenience or internet-based sampling, self-reported outcomes, and incomplete adjustment for confounding. The cross-sectional study assessment is shown in Table 3.

**Table 3:** NOS risk-of-bias assessment of cross-sectional studies NOS: Newcastle-Ottawa Scale

Study	NOS selection	NOS comparability	NOS outcome	Overall risk of bias
Guo et al. [17]	Low	Low	Low	Low to moderate
Marzo et al. [18]	Moderate	Moderate	Moderate	Moderate
Shiferaw et al. [19]	Moderate	Moderate	Low	Moderate
Estacio et al. [23]	Moderate	Moderate	Moderate	Moderate
Choi and Lee [24]	Moderate	Moderate	Low	Moderate
Marzo et al. [25]	High	Moderate	Moderate	Moderate to high
Nath et al. [26]	Moderate	Moderate	Moderate	Moderate

For non-cross-sectional studies, design-specific tools were used. The qualitative study was assessed using the CASP Qualitative Checklist, and the study conducted within a randomized trial framework was assessed using RoB 2. Measurement-validation studies were excluded from the main synthesis and risk-of-bias assessment. Table 4 presents the appraisal of non-cross-sectional studies included in the main synthesis.

**Table 4:** Risk-of-bias appraisal of non-cross-sectional studies included in the main synthesis CASP: Critical Appraisal Skills Programme; RoB 2: revised Cochrane Risk of Bias tool for randomized trials

Study	Study design	Appraisal tool used	Key domains assessed	Overall appraisal
Tieu et al. [20]	Qualitative interview study	CASP Qualitative Checklist	Study aim, appropriateness of qualitative design, recruitment strategy, data collection, researcher-participant relationship, ethical considerations, data analysis, clarity of findings, and relevance	Moderate
Miller et al. [21]	Study conducted within a randomized trial framework	RoB 2	Randomization process, deviations from intended intervention, missing outcome data, outcome measurement, and selection of the reported result	Moderate

Association Between Digital Health Literacy and Preventive Health Behaviors

Across the included studies, digital health literacy showed the clearest association with COVID-19 prevention and vaccination-related outcomes. Better eHealth or digital health literacy was linked with infection-prevention behaviors, preventive self-efficacy, and vaccination intention, suggesting a common pattern in which access, appraisal, and use of online health information supported prevention-related decisions during public health emergencies [17,24-26]. Evidence for broader preventive outcomes, including cancer screening decision support and chronic disease self-management, was more limited and came from fewer studies [20,21]. Table 5 shows prevention-related outcomes associated with digital or eHealth literacy across the included studies.

**Table 5:** Summary of evidence for digital health literacy and prevention-related outcomes

Study	Outcome	Direction of finding
Guo et al. [17]	COVID-19 preventive behaviors	Higher eHealth literacy was associated with better preventive behaviors
Choi and Lee [24]	COVID-19 preventive behaviors	Higher COVID-19-related eHealth literacy was associated with better preventive behaviors
Marzo et al. [25]	COVID-19 vaccination intention	Sufficient digital health literacy was associated with higher vaccination intention
Nath et al. [26]	COVID-19 vaccine uptake intention	eHealth literacy was positively associated with vaccine uptake intention
Miller et al. [21]	Colorectal cancer screening decision support	A low-literacy digital decision aid was feasible among vulnerable users
Tieu et al. [20]	Chronic disease self-management and portal-supported care	Portal use was relevant but affected by literacy, technical skill, and privacy concerns

**Figure 2:**
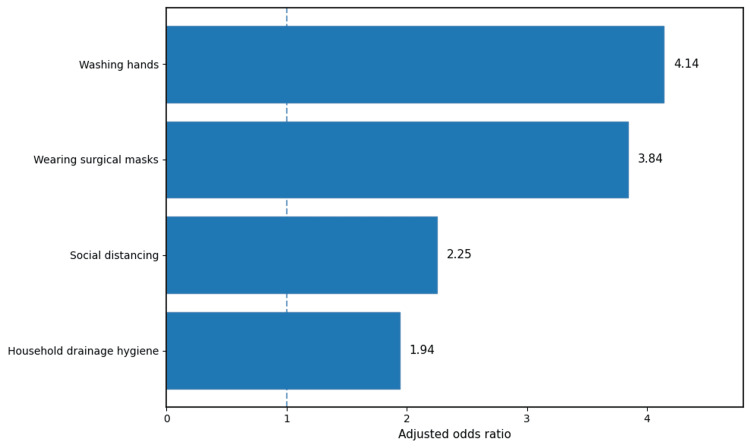
COVID-19 preventive behaviors associated with higher eHealth literacy

Figure 2 further illustrates the adjusted odds ratios for individual COVID-19 preventive behaviors associated with higher eHealth literacy in Guo et al. [17].

Socioeconomic and Digital Access Determinants of Digital Health Literacy

Socioeconomic and digital access factors were reported across several included studies. In the Ethiopia study among chronic disease patients, 46.5% of participants had high eHealth literacy. Higher education, higher income, daily internet use, positive attitudes toward online resources, and higher computer literacy were associated with higher odds of high eHealth literacy after adjustment [19]. Similar determinant patterns were reported in other settings. In Hong Kong, higher education and higher monthly income were associated with higher eHEALS scores [17]. In England, adequate health literacy was associated with internet access after adjustment, and lower education, lower income, older age, poor perceived health, and social isolation were related to lower internet access or health information use [23]. In the Malaysian B40 study, most participants reported ease in searching for COVID-19 information, but difficulties were reported in evaluating reliability, adding self-generated content, and protecting privacy [18].

**Figure 3:**
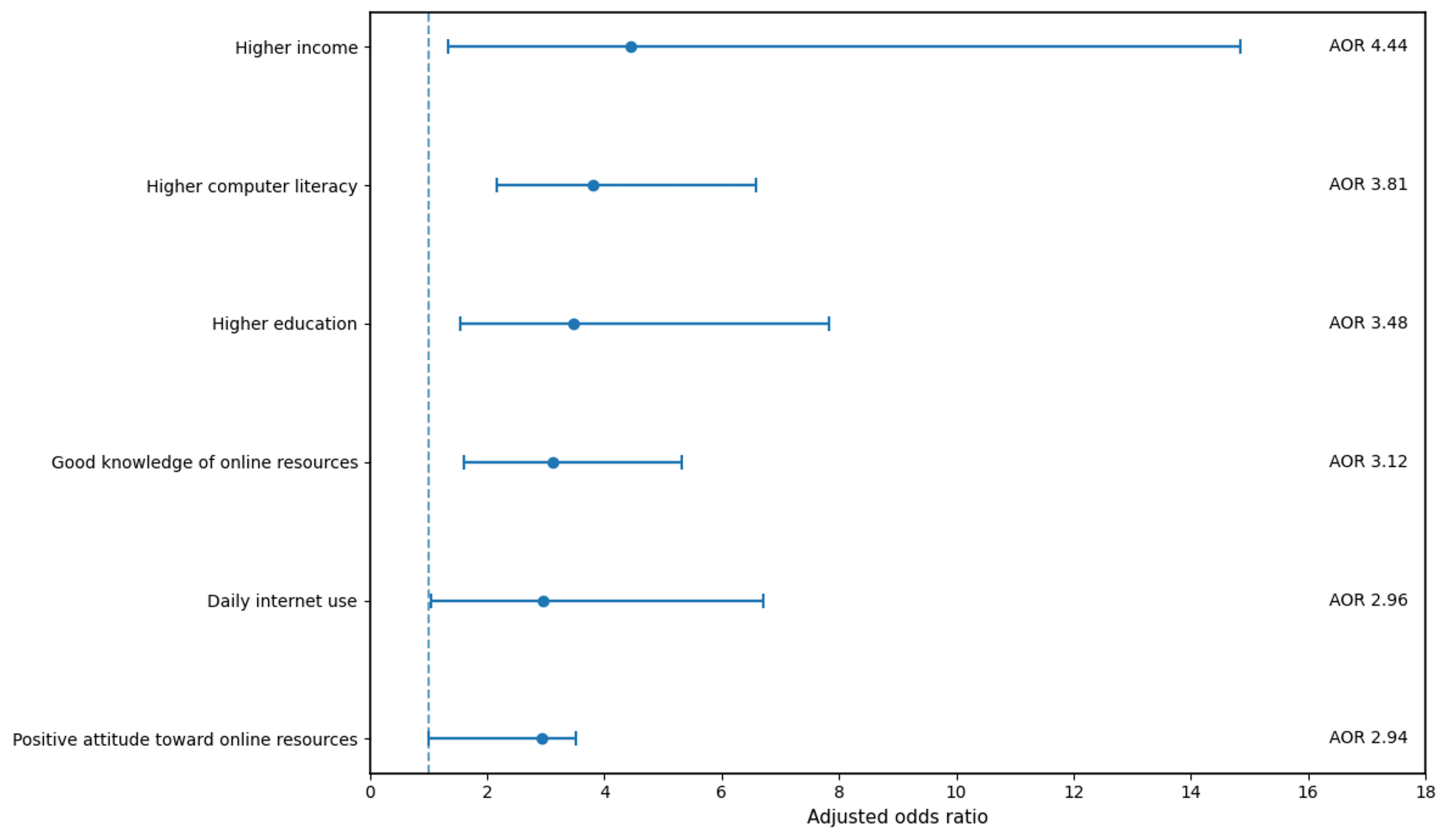
Predictors of high eHealth literacy in Ethiopia

Across these studies, the main reported determinants were education, income, internet access or use, computer literacy, health literacy, and information appraisal ability. Confounding adjustment varied across studies, with some studies reporting adjusted estimates and others presenting descriptive or subgroup findings [17-19,23]. Figure 3 presents the adjusted odds ratios for predictors of high eHealth literacy reported in the Ethiopia study among chronic disease patients [19].

Discussion

This systematic review identified that digital health literacy was associated with selected prevention-related outcomes, particularly COVID-19 preventive behaviors and vaccination intention, in studies that directly assessed these associations. Most evidence came from cross-sectional studies, so the findings should be interpreted as associations rather than causal effects. Across the quantitative studies that examined this relationship, higher digital or eHealth literacy was generally associated with better prevention-related behaviors, online health information use, or vaccination intention. Qualitative and usability studies provided supportive contextual evidence on barriers and facilitators to using digital tools but did not directly test statistical associations. The results suggest that digital health literacy is more than just a technical skill; it may be relevant to prevention because it involves finding credible information, assessing its relevance, and applying it to health decisions. This is particularly relevant in low-income and vulnerable communities where limited healthcare access, lower educational status, financial constraints, and restricted digital access may reduce opportunities for timely prevention.

The evidence suggests that gaps in digital prevention are not explained by individual ability alone. They are influenced by the design and delivery of digital systems. Miller and colleagues demonstrated that vulnerable patients would be able to complete a mobile health iPad application if it provided simple navigation, low reading level information, large buttons to click, and audio narration [21]. This finding suggests that vulnerable users may benefit from digital health tools when design features are adapted to literacy, usability, and access needs. Digital exclusion may therefore reflect inadequate design, limited user support, and failure to adapt technologies to users' literacy, access, and usability needs.

The measurement aspects of digital health literacy are also significant in interpreting digital health literacy. Paige et al. demonstrated that electronic health literacy is composed of awareness of online health resources, information-seeking capacity, and online information evaluation and action [22]. The present review's findings are supported by this multidimensional perspective, but behavioral interpretation was based only on studies that directly assessed prevention-related outcomes rather than on psychometric evidence alone. Studies that assessed digital health literacy domains indicated that skills such as evaluating reliability, determining relevance, and applying online information may be important for prevention-related decision-making [18]. Estacio et al. also showed that social determinants such as age, income, education, perceived health, and social isolation were associated with health literacy, internet access, and online health information seeking [23]. Accordingly, the present review interpreted socioeconomic position and digital access as contextual factors related to digital health literacy and online health information use, rather than as direct evidence of preventive behavior.

Studies conducted during the COVID-19 pandemic showed the strongest association between digital health literacy and prevention-related outcomes. This may be because the pandemic created an urgent need for people to rapidly access, interpret, and apply online health information for immediate protective decisions, such as mask use, social distancing, hand hygiene, and vaccination. In this context, digital health literacy was closely connected to time-sensitive prevention decisions and public health guidance. COVID-19-related eHealth literacy and self-efficacy were associated with preventive behaviors among young adults [24]. Digital health literacy was also associated with intention to vaccinate in a multi-country adult sample [25]. Among young adults in a lower-middle-income country, eHealth literacy was positively related to vaccine uptake intention, although vaccine hesitancy remained an important barrier [26]. Thus, the stronger pandemic-related associations may reflect the high dependence on digital information during COVID-19, rather than a causal effect of digital health literacy alone. The results suggest that digital health literacy could play a role in the promotion of preventive actions by increasing self-efficacy in the ability to trust credible information and decreasing uncertainty during public health crises.

The findings have important implications for digital public health equity. Azzopardi-Muscat and Sørensen proposed that in a fair digital public health era, the focus should be on health literacy [27]. This viewpoint is particularly relevant to low-income communities as digital prevention programs, which may arguably exacerbate inequities by taking a blanket approach, would require everyone to have access to the internet, a high reading level, digital confidence, and trust in online systems. Kim et al. had an emphasis on social determinants of health such as income, education, race, language, geography, and healthcare access as being important to digital health equity in cardiovascular medicine [28]. The current review substantiates this claim by demonstrating that digital health literacy is not an individual-level phenomenon only, but a phenomenon that also depends on structural factors.

The conclusions of the review are supported by broader evidence. Yuen et al. found that digital health literacy is linked to sociodemographic characteristics, health resource utilization, and health outcomes [29]. More and more attention is being paid to the concept of digital health literacy by scholars in public health, health communication, and medical informatics, as Yang et al. demonstrated [30]. This ever-expanding literature is useful, albeit somewhat inconsistent. A large number of the studies that are available are cross-sectional, self-reported, and COVID-19-centric. Evidence continues to be limited for preventive behaviors that do not include the pandemic, including cancer screening, cardiovascular risk reduction, diabetes prevention, and routine immunization. Papp-Zipernovszky et al. also demonstrated that there is an association between digital health literacy generation gaps and health information seeking and health empowerment [31]. This suggests that age differences in digital confidence, platform preference, and information appraisal should be taken into consideration for future interventions.

In general, this review indicates that digital health literacy may help reduce the digital prevention divide when digital health tools are accessible, trusted, and socially appropriate. Digital health strategies for low-income and vulnerable communities should consider affordability, internet connectivity, ease of use, language, privacy issues, misinformation, and user support. The limitations related to cross-sectional designs, self-reported outcomes, intention-based measures, and possible exclusion of digitally marginalized populations through online recruitment have been consolidated in the Limitations section. Future studies should prioritize longitudinal and intervention designs, include observed preventive behaviors where possible, and intentionally recruit digitally marginalized populations.

Limitations

Most included studies were cross-sectional and relied on self-reported or intention-based outcomes, limiting causal interpretation and verification of actual behavior change. The evidence was largely focused on COVID-19 prevention, with limited data on routine vaccination, cancer screening, chronic disease prevention, and other non-pandemic outcomes. Heterogeneity in study populations, digital health literacy measures, socioeconomic definitions, and outcome assessments prevented meta-analysis. Internet-based recruitment may have excluded individuals with the greatest digital disadvantage, including those without reliable internet access, devices, or technical skills. The inclusion of only full-text English-language studies may have introduced language and publication bias. The small number of eligible studies and the overall moderate risk of bias also limit the generalizability of the findings.

## Conclusions

In this review, digital health literacy was generally associated with selected prevention-related outcomes, including COVID-19 prevention behaviors, intention to vaccinate, and online health information use. This evidence suggests that individuals with higher digital health literacy may be better able to find health information, assess its trustworthiness, and use it to support prevention-related decisions, such as getting vaccinated, practicing infection control, screening for diseases, or managing chronic conditions. The results also reveal that the digital prevention gap is complex, influenced by socioeconomic disadvantage, limited access to the internet, lower health literacy, lower computer skills, concerns about privacy, and exposure to misinformation. Thus, digital prevention strategies could exacerbate current inequities if they only provide information online and fail to consider the issues of usability, trust, language, affordability, and user support. Equitable digital health interventions should consider the needs of users with low literacy and low resources, accessibility, cultural relevance, privacy protection, and guided use. Future studies should focus on longitudinal and intervention studies with actual preventive behaviors and the inclusion of digitally marginalized groups that are not usually reached through online surveys.
